# Identification and validation of a ferroptosis‐related gene signature for predicting survival in skin cutaneous melanoma

**DOI:** 10.1002/cam4.4706

**Published:** 2022-04-04

**Authors:** Shuai Ping, Siyuan Wang, Yingsong Zhao, Jinbing He, Guanglei Li, Dinglin Li, Zhuo Wei, Jianghai Chen

**Affiliations:** ^1^ Department of Orthopaedics, Liyuan Hospital, Tongji Medical College Huazhong University of Science and Technology Wuhan China; ^2^ Department of Integrated Traditional Chinese and Western Medicine, Liyuan Hospital Tongji Medical College of Huazhong University of Science and Technology Wuhan China; ^3^ Department of Hand Surgery, Union Hospital, Tongji Medical College Huazhong University of Science and Technology Wuhan China

**Keywords:** ferroptosis, gene signature, melanoma, overall survival, tumor immunity

## Abstract

**Purpose:**

Ferroptosis plays a crucial role in the initiation and progression of melanoma. This study developed a robust signature with ferroptosis‐related genes (FRGs) and assessed the ability of this signature to predict OS in patients with skin cutaneous melanoma (SKCM).

**Methods:**

RNA‐sequencing data and clinical information of melanoma patients were extracted from TCGA, GEO, and GTEx. Univariate, multivariate, and LASSO regression analyses were conducted to identify the gene signature. A 10 FRG signature was an independent and strong predictor of survival. The predictive performance was assessed using ROC curve. The functions of this gene signature were assessed by GO and KEGG analysis. The statuses of low‐risk and high‐risk groups according to the gene signature were compared by GSEA. In addition, we investigated the possible relationship of FRGs with immunotherapy efficacy.

**Results:**

A prognostic signature with 10 FRGs (CYBB, IFNG, FBXW7, ARNTL, PROM2, GPX2, JDP2, SLC7A5, TUBE1, and HAMP) was identified by Cox regression analysis. This signature had a higher prediction efficiency than clinicopathological features (AUC = 0.70). The enrichment analyses of DEGs indicated that ferroptosis‐related immune pathways were largely enriched. Furthermore, GSEA showed that ferroptosis was associated with immunosuppression in the high‐risk group. Finally, immune checkpoints such as PDCD‐1 (PD‐1), CTLA4, CD274 (PD‐L1), and LAG3 were also differential expression in two risk groups.

**Conclusions:**

The 10 FRGs signature were a strong predictor of OS in SKCM and could be used to predict therapeutic targets for melanoma.

## Graphical abstract

The signature was independently correlated with OS in both the training and validation cohorts, improving the prediction of SKCM prognosis. FRGs signature could predict the expression level of ICIs and be applied to guide immunotherapy strategies.

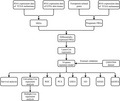

## 1 INTRODUCTION

Malignant melanoma, which is increasing in incidence worldwide, is the most aggressive form of skin cancer. There were approximately 106,110 new cases of melanoma and 7180 deaths from melanoma in the United States in 2021.^1^ Over the last two decades, the range of available systemic treatment options for melanoma has expanded and include immunotherapy (CTLA‐4, PD‐1, LAG‐3, and PD‐L1 inhibitors), MEK, and targeted therapy (BRAF inhibitors).^2^, ^3^, ^4^, ^5^, ^6^ However, the prognosis of advanced or metastatic melanoma is poor,^7^ and the demand for novel biomarkers to improve melanoma early diagnosis and prognosis has increased in the recent years.^8^ Moreover, despite improvements in staging, the prognosis of advanced melanoma is heterogeneous, with high variability in the overall estimate among stages III and IV.^9^, ^10^ Thus, the development of novel signatures that can significantly improve melanoma diagnosis and increase the accuracy of predicting melanom prognosis is needed.

Ferroptosis is a form of programmed cell death caused by iron‐dependent lipid peroxidation^11^ and has a crucial role in inhibiting tumor cell proliferation, invasion, and metastasis.^12^ Ferroptosis can trigger immune responses, especially in malignant tumors resistant to conventional therapies.^13^, ^14^, ^15^ Ferroptosis has a dual role in cancer because ferroptotic tumor cells release some signaling molecules that either promote or inhibit the tumor proliferation^16^, ^17^, ^18^; however, the role of these molecules in tumor is incompletely understood.^16^ A current research has demonstrated that lymph protects melanoma from ferroptosis, increases melanoma cell viability during metastasis, and increases the formation of distant metastasis, providing new perspectives for studying metastasis.^19^ Therefore, identifying ferroptosis‐related biomarkers for the prognosis of skin cutaneous melanoma (SKCM) is essential.

Some studies identified prognostic signatures based on FRGs in tumors based on public databases.^20^, ^21^ In this respect, a 19‐gene signature predicted glioma cell death and prognosis,^20^ and a 10‐gene signature predicted OS in hepatocellular carcinoma.^21^ However, no studies have identified ferroptosis‐related signature that can predict OS in patients with SKCM. To identify and validate a prognostic signature and improve the diagnosis and therapy of SKCM, TCGA, GTEx, and GEO databases were comprehensively searched for novel and previously identified FRGs. The functions of this signature in the tumor microenvironment were assessed through enrichment analysis.

## 2 MATERIALS AND METHODS

### 2.1 Data acquisition and processing

The RNA‐seq expression profiles of SKCM and normal skin tissues were obtained from TCGA and GTEx databases, respectively.^22^ The GSE65094 gene expression dataset and follow‐up clinical information were downloaded from the GEO. Read counts were normalized through scale. The TCGA‐SKCM dataset (471 samples) and GSE65094 dataset (214 samples) were selected as the training and validation sets, respectively. Then, 253 FRGs were obtained from the FerrDb database.^23^

### 2.2 Analysis of DEGs

To identify significant DEGs in melanoma, RNA‐seq data extracted from TCGA (471 SKCM samples and one normal skin sample) and the GTEx database (812 normal skin samples) were normalized by log_2_‐transformed FPKM and merged into one dataset for subsequent analysis. DEGs were determined by “limma” package in R.

### 2.3 Signature construction and validation

The correlation between FRGs and OS in TCGA training set was analyzed by univariate Cox regression analysis using the “survival” package. FRGs with *p* < 0.01 were considered to have prognostic significance. Differentially expressed FRGs were identified by overlapping prognostic FRGs and DEGs. A gene signature with 10 FRGs was selected by LASSO regression.^24^, ^25^ LASSO estimates were based on 1000‐fold cross‐validation to avoid overfitting. In this model, risk scores were calculated via multiplying the expression values of 10 FRGs by their regression coefficients (A), as follows: risk score = (expression of gene 1 × A^1^ of gene 1) + (expression of gene 2 × a A^2^ of gene 2) + (expression of gene N × A^N^ of gene N).

Patients were divided into a low‐ and a high‐risk group based upon median risk score. Survival status were displayed in the Kaplan–Meier curve. The model predictive ability was assessed using heat maps, forest plots, risk score maps, OS curves, and ROC curves. Based on prognostic ferroptosis‐related DEGs, PCA, and t‐SNE were generated using the “stats” and “Rtsne” package of R. External validation of the signature was performed using the GSE65904 dataset. This dataset was divided into a high‐ and low‐risk group based upon the cutoff value of the signature. OS curves were compared between these two groups using Kaplan–Meier analysis to validate the signature.

### 2.4 Gene set enrichment analysis

GO and KEGG analyses of FRGs differentially expressed were performed using “clusterProfiler” package in R. GSEA of GO and KEGG gene sets was performed using the GSEA software version 4.1.0. The number of random sample permutations in each analysis was set to 1000.

### 2.5 Immune correlation analysis

The ssGSEA was performed to assess the tumor‐infiltrating immune cell subsets and immune‐related functions.^21^ The expression of immune checkpoint gene might predict therapeutic effect of immune checkpoint inhibitors (ICIs).^26^ Thus, we investigated four ICIs: PD‐1 and PD‐L1, CTLA‐4, and LAG‐3 in melanoma.^27^ We also estimated the relationship between ICIs and risk score through the spearman correlation analysis, which intended to assess potential application of FRGs signature in immunotherapy.

### 2.6 Validation

A study of single cell malignant melanoma transcriptomes defined two main transcriptional states of melanoma cells: the MITF and AXL gene programs.^28^ We choose the A2058 (MITF) and A375 (AXL) cell lines for our study. Human melanoma cell lines (A2058, A375) and human epithelial cell line HaCaT were purchased from the Shanghai Zhong Qiao Xin Zhou Biotechnology Co.,Ltd. These three cell lines were cultured in DMEM (high‐glucose) medium (Gibco) containing 10% FBS (Gibco) at 37°C with 5% CO_2_ in an incubator. Total RNA of HaCaT, A2058, and A375 cells were extracted using RNA‐easy kit (Vazyme). The primers used in this study were synthesized by GENECREATE (WUHAN GENECREATE BIOLOGICAL ENGINEERING, LTD) (Table 1). Following, the reverse transcription was conducted with the HiFiScript cDNA synthesis kit (Vazyme) to generate cDNA. The qPCR was performed using an LineGene 9600 Plus instrument (Bioer Technology) and 2× SYBR Green Qpcr MasterMix (SEVEN BIOTECH). The CT values were normalized to the expression of the endogenous housekeeping gene GAPDH, and the 2(−ΔΔCt) values were calculated for relative quantification. The reactions were performed in triplicate. The comparisons among multiple groups were conducted by one‐way ANOVA. Statistical analyses were carried out using GraphPad Prism 9.0.0 software.

**TABLE 1:** Primer sequence for qPCR

Gene	Forward sequence (5′–3′)	Reverse sequence (5′–3′)
CYBB	GTCAAGTGCCCAAAGGTGTC	TCTGTCCAGTCCCCAACGAT
IFNG	GCAGGTCATTCAGATGTAGCG	GTCTTCCTTGATGGTCTCCAC
FBXW7	CTGGGCTTGTACCATGTTCAG	GGACAGATGTAATTCGGCGTC
ARNTL	AGAGGTGCCACCAATCCATAC	CGGTCACATCCTACGACAAAC
PROM2	AGAACGGCGAGCTCTTTGAG	CTGCTGATAGGCTTGGTGGAT
GPX2	CAGTCTCAAGTATGTCCGTCCTG	CTCGTTCTGCCCATTCACCT
JDP2	GAGGAAGAGGAGCGAAGGAA	GTGTCGGTTCAGCATCAGGA
SLC7A5	CCGTGGACTTCGGGAACTAT	GTGAACAGGGACCCATTGAC
TUBE1	AGAGTGGTTGGTGATGGTGG	TCTCAGTGGTCCCTGCAGAA
HAMP	TGACCAGTGGCTCTGTTTTCC	TACGTCTTGCAGCACATCCC

## 3 RESULTS

### 3.1 Identification of DEGs in SKCM

**FIGURE 1:**
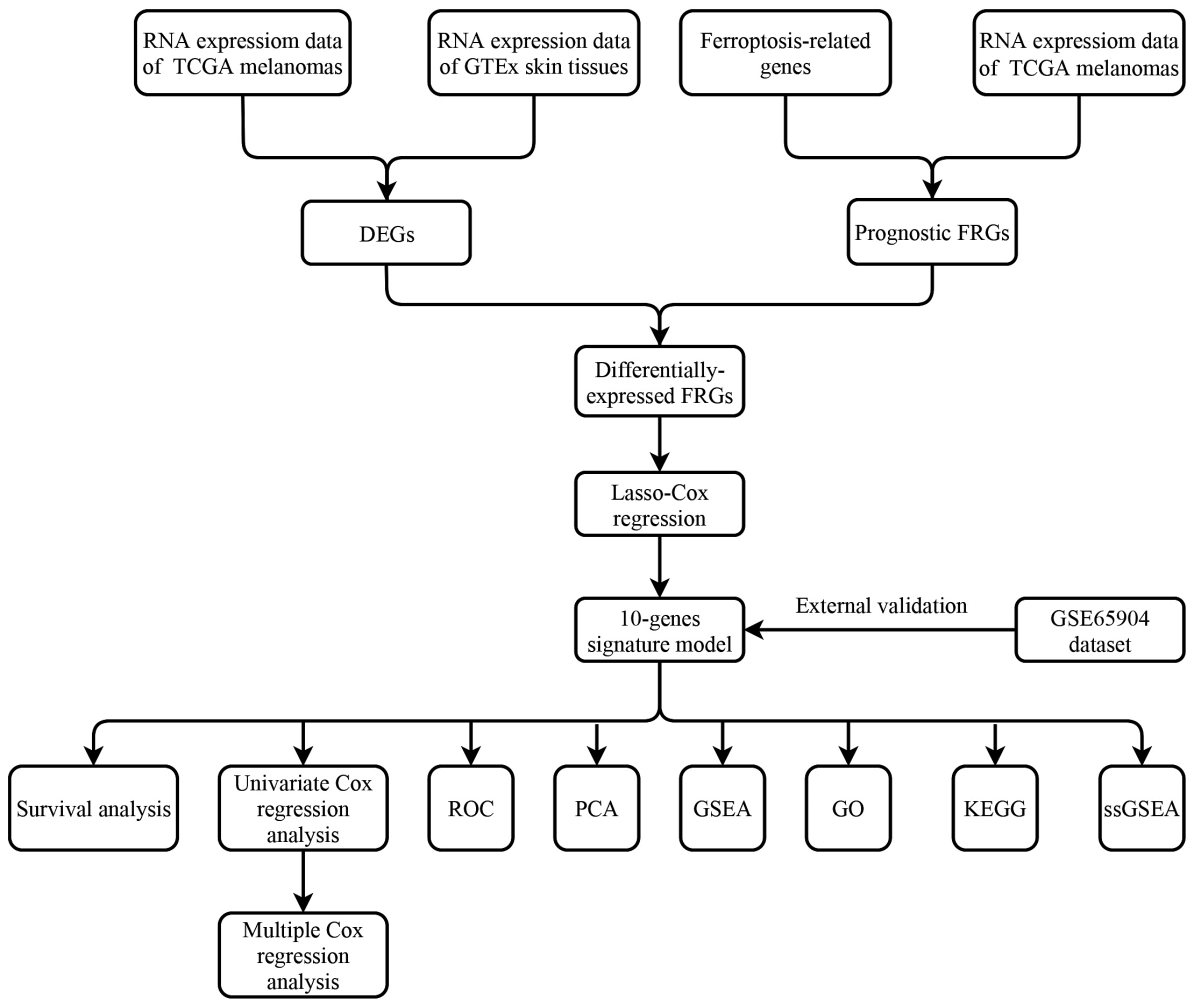
The process flow of this study

The total 104 FRGs were identified to match the RNA‐seq data from the TCGA. Using “limma” package with an absolute log_2_‐fold change (FC) >1 and an adjusted *p* < 0.05, 24 DEGs (17 upregulated and seven downregulated) and 16 DEGs (13 upregulated and three downregulated) related to ferroptosis and iron metabolism in SKCM were identified in TCGA and ICGC, respectively (Figure 1).

### 3.2 Screening of differentially expressed FRGs

**FIGURE 2:**
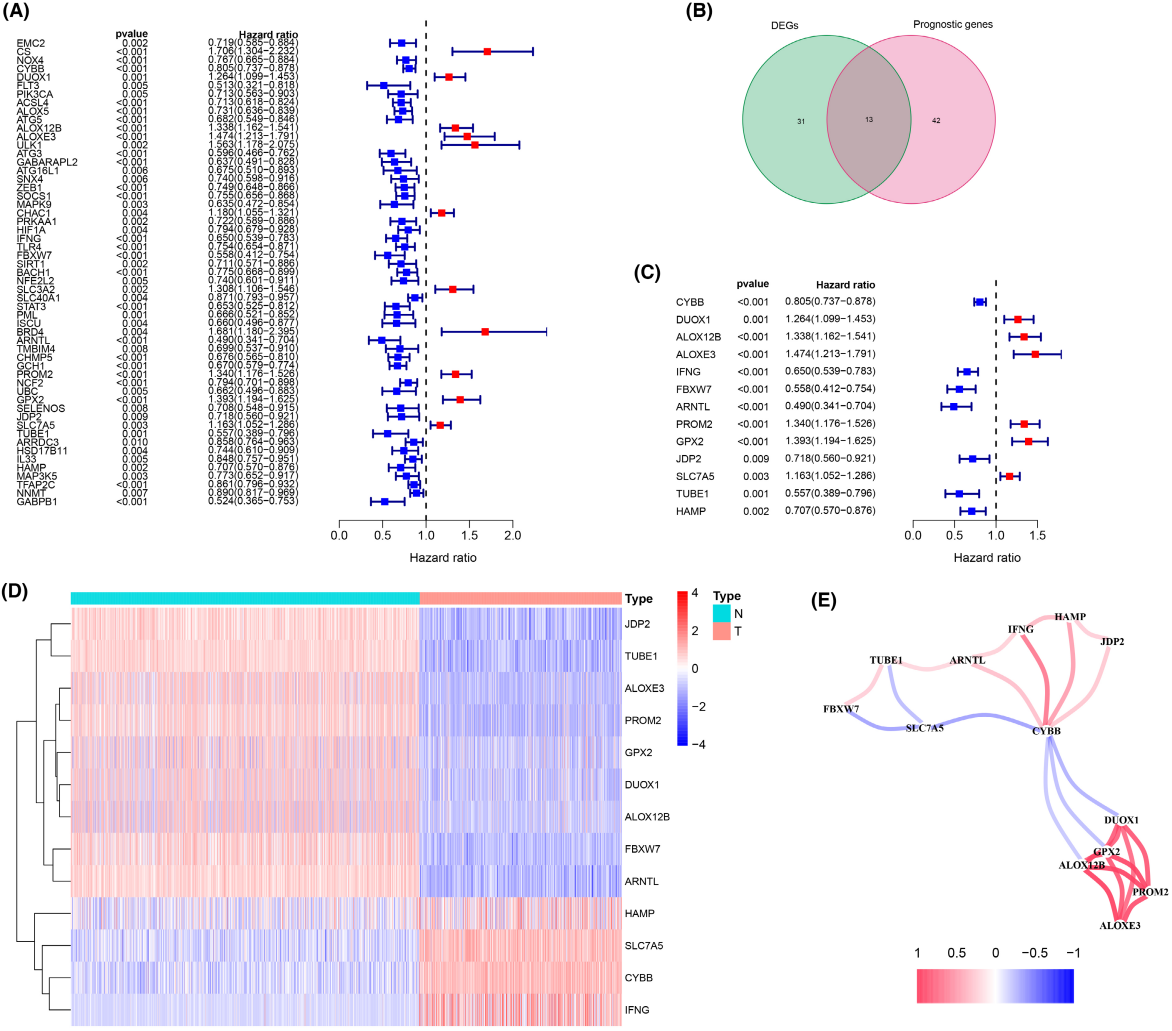
Identification of the prognostic ferroptosis‐related DEGs in the TCGA. (A) Forest plot of the prognostic ferroptosis‐related gene (FRGs) of skin cutaneous melanoma in the TCGA. (B) Venn diagram of the overlapped genes between DEGs and FRGs. (C) Forest plots showing the 13 overlapping genes of the prognostic ferroptosis‐related DEGs. (D) A heat map of the 13 prognostic ferroptosis‐related DEGs, four genes of which were upregulated, and the remaining nine genes of which were downregulated in tumor tissue. (E) The correlation network of 13 prognostic ferroptosis‐related DEGs. The correlation coefficients are represented by different colors

The comparison of RNA‐seq data of melanoma samples (*n* = 471) and normal skin samples (*n* = 813) in the TCGA and GTEx databases identified 44 DEGs with an absolute log_2_‐FC > 1 and an adjusted *p* < 0.05 (FC ≥ 1, FDR ≤ 0.05) (Figure 1). The total 253 FRGs were obtained from FerrDb database. The univariate Cox proportional hazard regression analysis showed that 55 FRGs were significant predictors of OS (*p* <0.01) (Figure 2A). Of these, 13 FRGs were differentially expressed (four upregulated and nine downregulated) in tumor tissues (Figures 2B–D). The relationship in these genes is displayed in Figure 2E.

### 3.3 Construction of a prognostic model using the TCGA dataset

A prognostic signature with 10 FRGs (CYBB, IFNG, FBXW7, ARNTL, PROM2, GPX2, JDP2, SLC7A5, TUBE1, and HAMP) was identified by LASSO Cox regression. The risk score was calculated as follows:

Risk score = (0.183 × expression level of PROM2) + (0.057 × expression level of GPX2) + (0.020 × expression level of SLC7A5) + (−0.029 × expression level of CYBB) + (−0.222 × expression level of IFNG) + (−0.242 × expression level of FBXW7) + (−0.202 × expression level of ARNTL) + (−0.061 × expression level of JDP2) + (−0.127 × expression level of TUBE1) + (−0.084 × expression level of HAMP).

**FIGURE 3:**
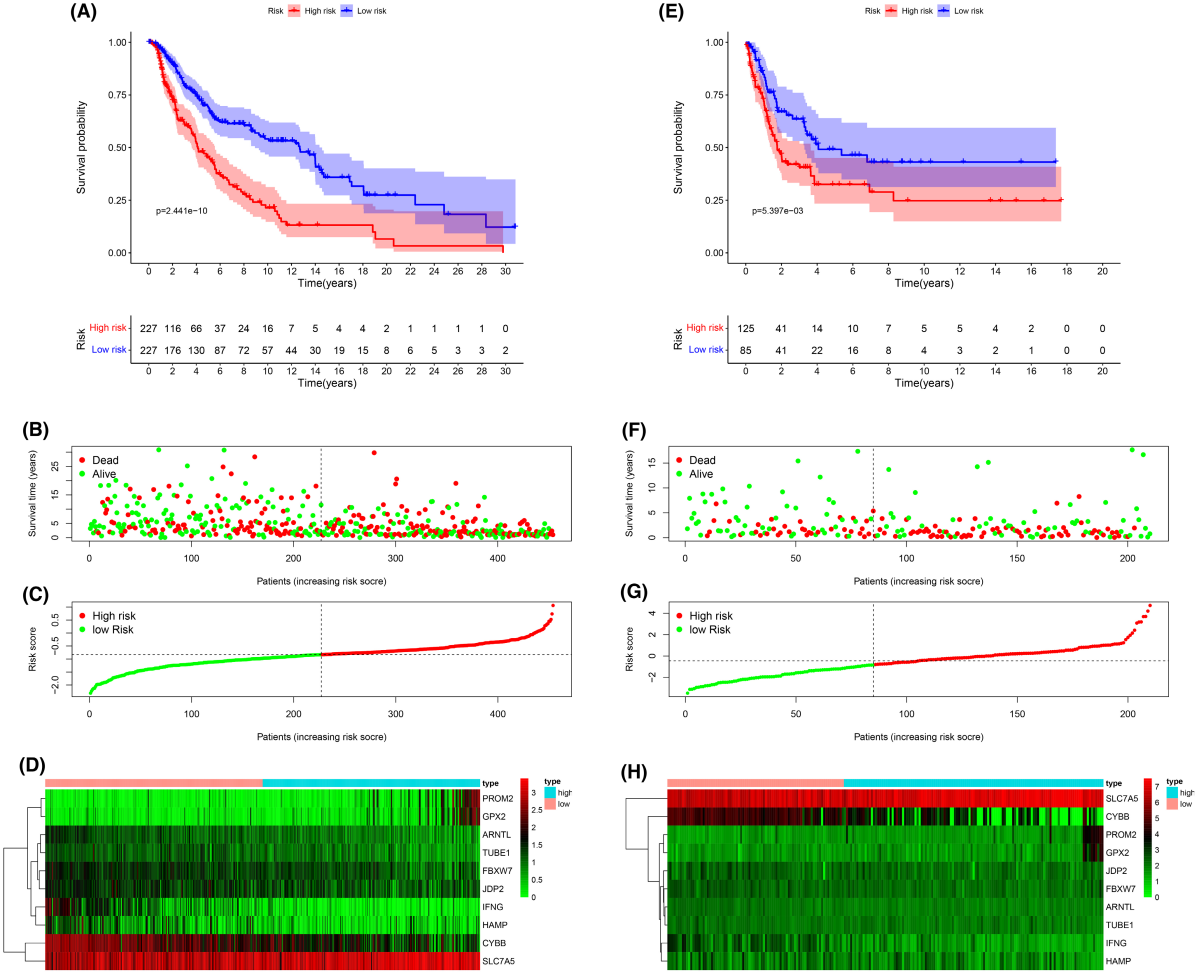
Kaplan–Meier curves for overall survival in the high‐risk and low‐risk groups. (A) The training dataset, (E) validation dataset. The survival status, risk score distribution, and risk genes expression in the datasets. (B, C, D) Training dataset, (F, G, H) validation dataset. (green and red lines/dots represent low and high risk, respectively)

**FIGURE 4:**
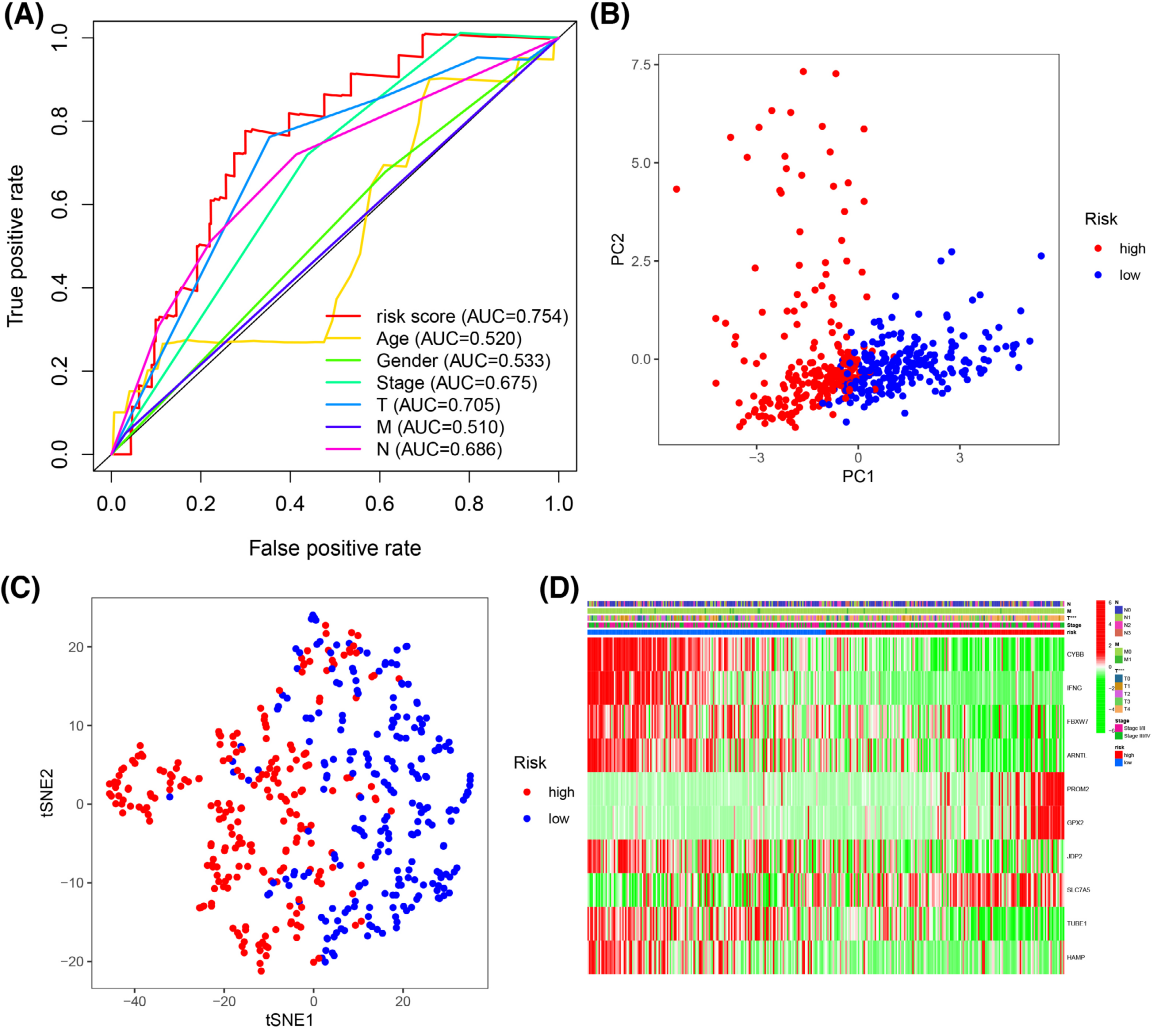
(A) The AUC for risk score and clinical features according to the ROC curves in the training dataset. Clinical feature: Age, gender, stage, and T, N, M stage. (B) PCA plot of the training dataset. (C) The t‐SNE analysis of the training dataset. (D) The differential expression of ferroptosis‐related genes in different clinical features. ***p < 0.001

The patients were divided into a high‐risk and a low‐risk group according to the median cutoff value. The Kaplan–Meier analysis indicated that prognosis was significantly worse in the high‐risk group than in the low‐risk group in the training dataset (Figure 3A, *p* < 0.01). The risk score distribution plot and survival status curves indicated that the high‐risk group from this dataset had lower survival (Figure 3B) and higher risk scores (Figure 3C). Moreover, as the risk score increased, the expression of protective genes (CYBB, FBXW7, IFNG, ARNTL, JDP2, TUBE1, and HAMP) decreased, whereas the expression of risk genes (PROM2, GPX2, and SLC7A5) increased in this dataset (Figure 3D). Similar results were obtained in the validation dataset (Figures 3E–H). The AUC value of the risk score (0.754) was higher than those of clinical indicators, such as gender, age, and TNM stage (Figure 4A). PCA and t‐SNE analyses revealed that samples were distributed in two principal components (Figures 4B,C). The FRGs genes were differentially expressed between different T stages (Figure 4D, *p* < 0.001).

### 3.4 Independent prognostic value of the gene signature

**FIGURE 5:**
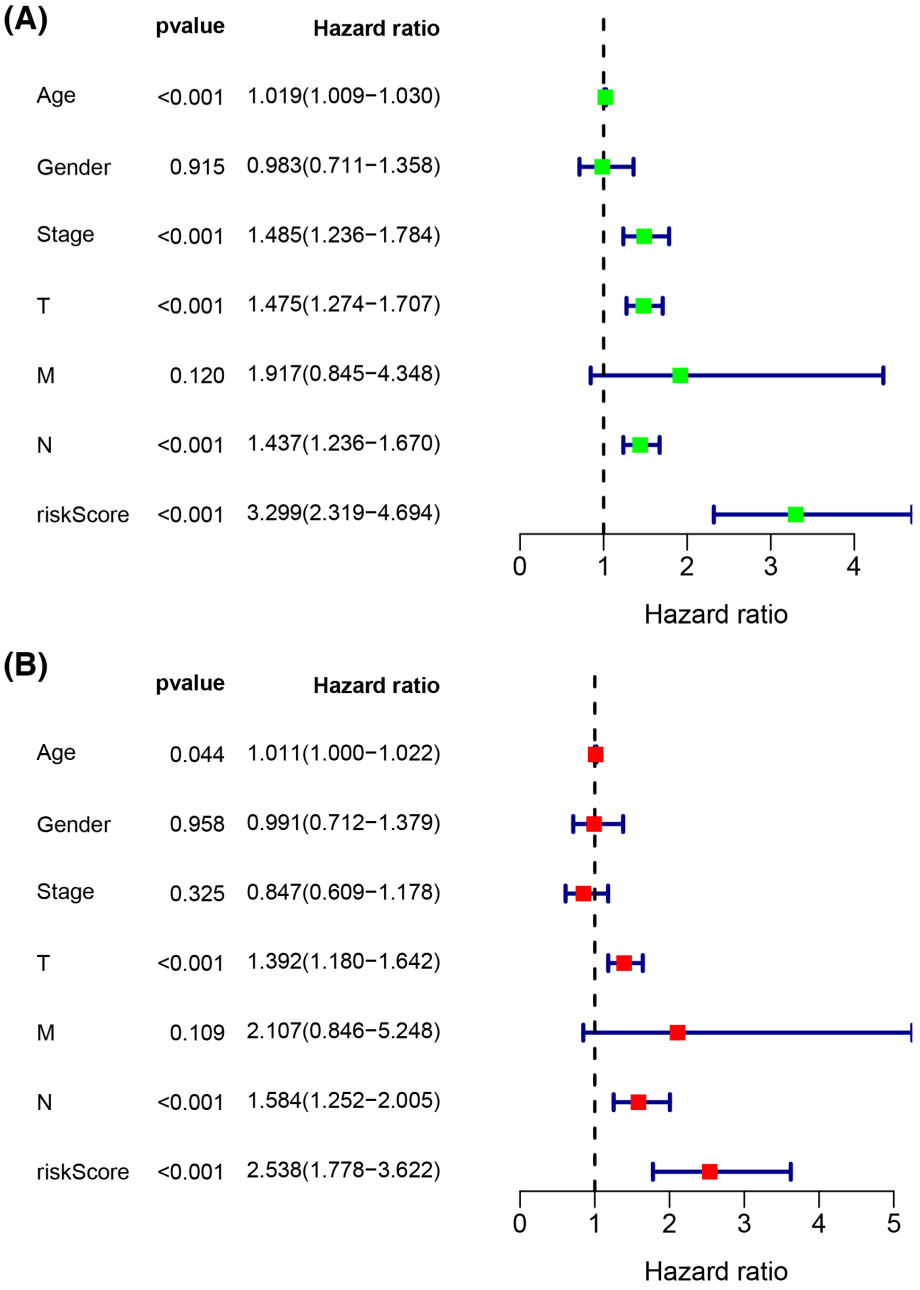
Forest plot of (A) the univariate and (B) multivariate Cox regression analysis showing that the age, T stage, N stage, and risk score were independent prognostic predictors

Univariate and multivariate Cox regression analyses of clinical characteristics (age, gender, stage, risk score, and TNM stage) in TCGA were performed to determine whether the risk score was an independent predictor of OS. In the univariate Cox regression analysis, the risk score was significantly correlated with OS in the training dataset (HR = 3.299, 95% CI = 2.319–4.694, *p* < 0.001) (Figure 5A). Multivariate Cox regression analysis indicated that age (HR = 1.011, 95% CI = 1.000–1.022, *p* = 0.044), T stage (HR = 1.392, 95% CI = 1.180–1.642, *p* < 0.001), N stage (HR = 1.584, 95% CI = 1.252–2.005, *p* < 0.001), and the risk score of the prognostic signature (HR = 2.538, 95% CI = 1.778–3.622, *p* <0.001) were independent predictors of OS (Figure 5B).

### 3.5 Functional enrichment analysis

**FIGURE 6:**
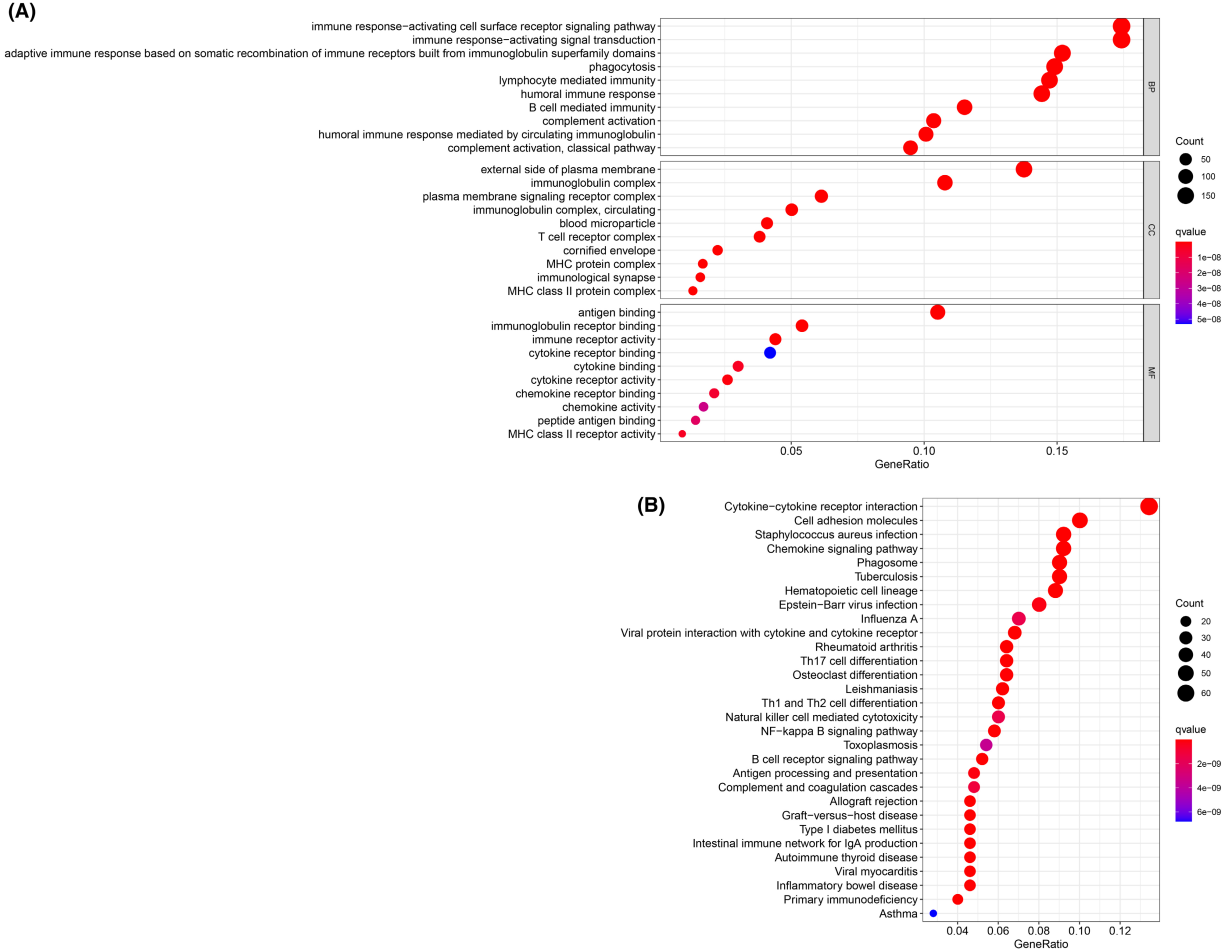
GO and KEGG enrichment analysis of DEGs. (A) GO enrichment analysis of the DEGs. (B) KEGG enrichment analysis of the DEGs

**FIGURE 7:**
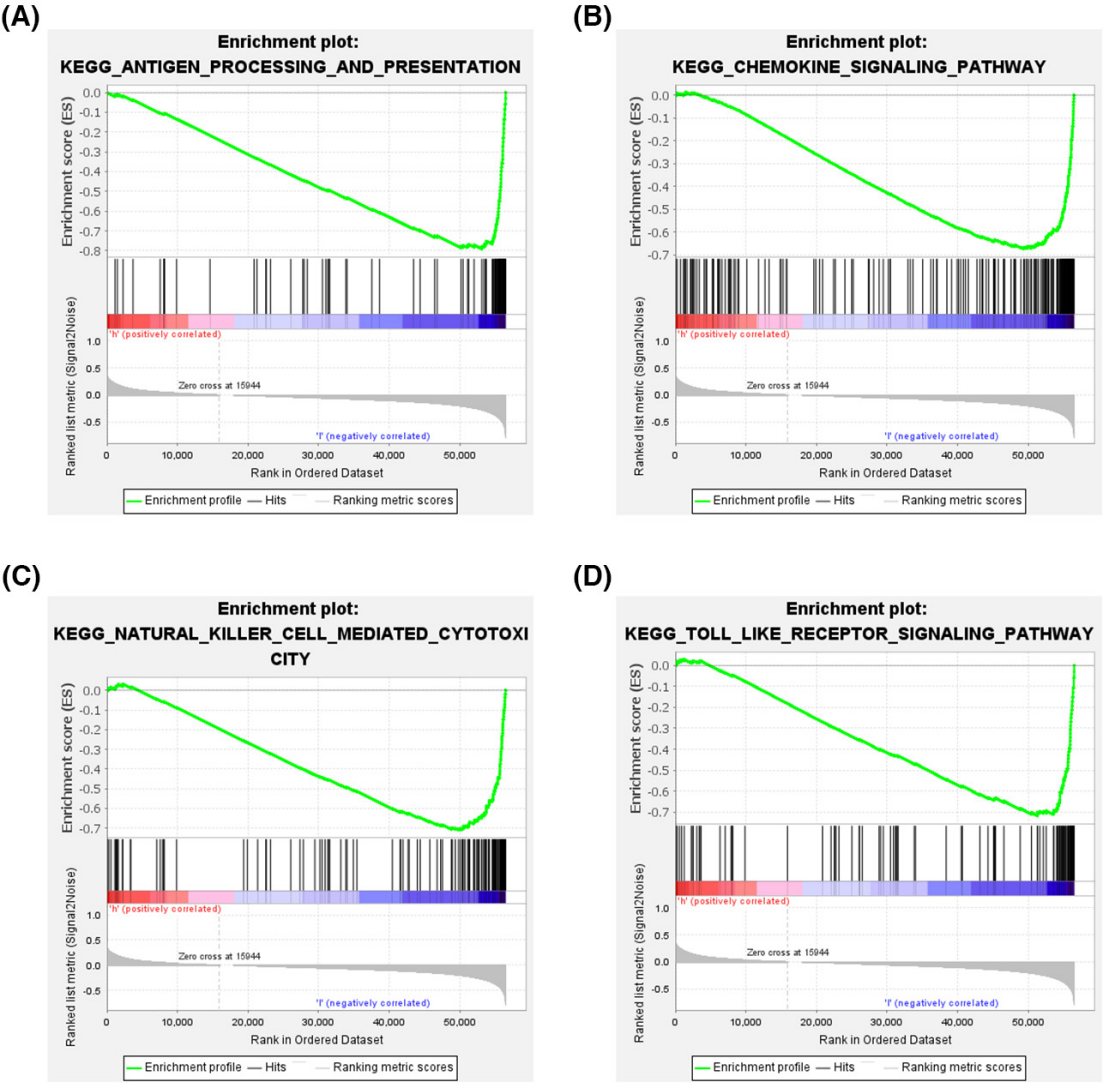
Analysis of enriched pathways. KEGG analysis (A–D) of Gene Set Enrichment Analysis in the low‐risk groups in skin cutaneous melanoma

GO and KEGG enrichment analyses of risk genes differentially expressed between the high‐risk and low‐risk groups were performed to assess the biological functions and pathways correlated with risk scores. GO analysis indicated that the DEGs were enriched in immune‐related biological processes, including immune response‐activating cell surface receptor signaling pathway, and immune response‐activating signal transduction; and immune‐related molecular functions, such as antigen binding, and immunoglobulin receptor binding; and immune‐related cell components, such as external side of plasma membrane and immunoglobulin complex (Figure 6A). KEGG pathway analysis showed that the DEGs were enriched in ferroptosis‐related immune pathways, such as cytokine‐cytokine receptor interaction, cell adhesion molecules, chemokine signaling pathway, and phagosome (Figure 6B). GSEA indicated that genes in low‐risk group were significantly enriched in immune pathways, including Toll‐like receptor signaling pathway, chemokine signaling pathway, natural killer cell‐mediated cytotoxicity, and antigen processing and presentation (Figure 7A–D). These results suggest a ferroptosis‐related immunosuppressive status in high‐risk group.

### 3.6 Immunity and gene expression

**FIGURE 8:**
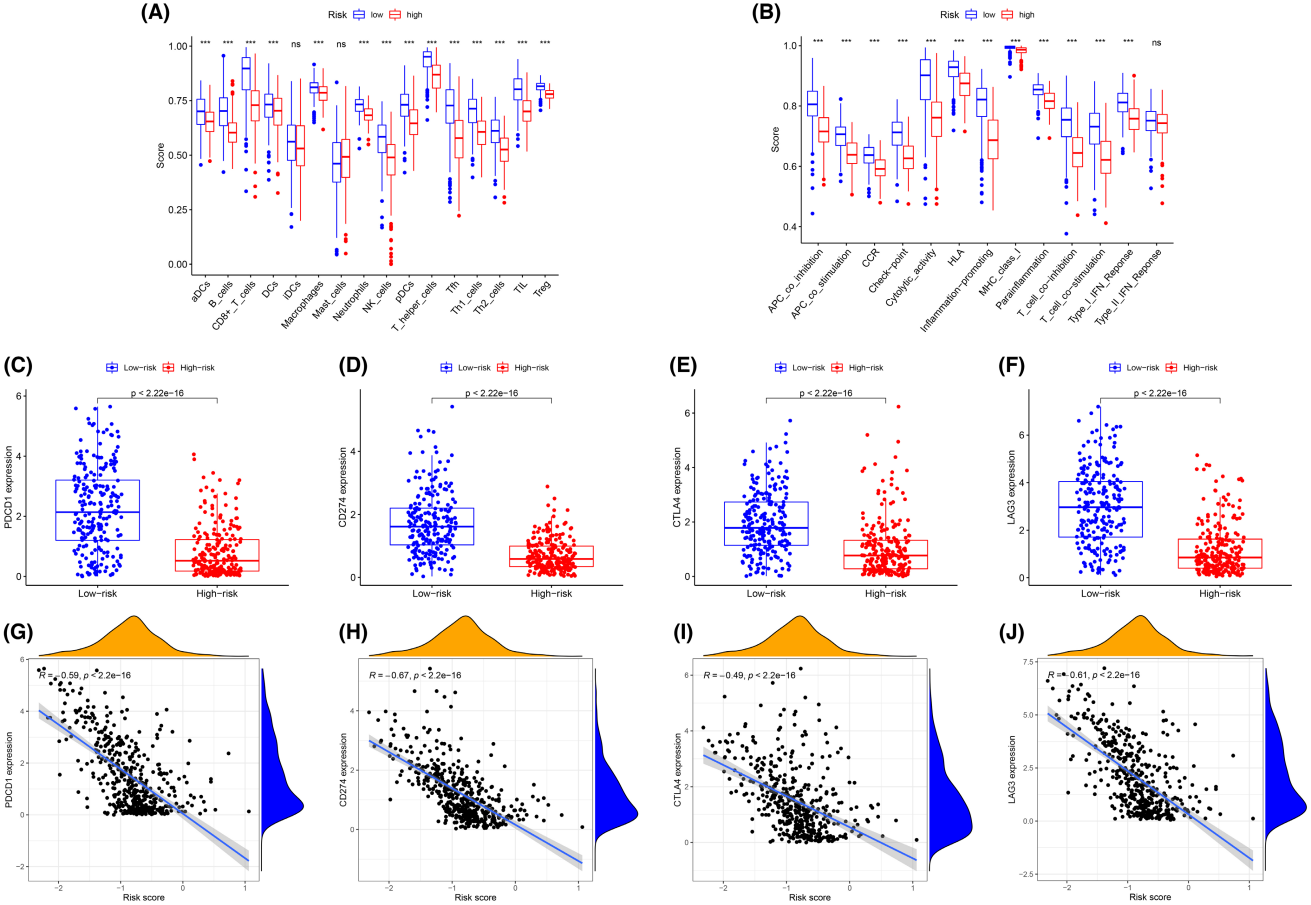
Comparison of the immune cell subpopulations and related functions between the different risk groups: (A) Scores of the 16 immune cells and (B) Scores of the 13 immune‐related functions. (C–F) The comparison of the expression levels of PDCD1 (PD‐1), CD274 (PD‐L1), CTLA‐4, and LAG‐3 between high‐risk and low‐risk groups. (G–J) Significant negative association between the risk score and ICB receptors PDCD‐1 (*R* = −0.59, *p* < 0.001, H), CD247 (*R* = −0.67, *p* < 0.001), CTLA4 (*R* = −0.49, *p* < 0.001), and LAG3 (*R* = −0.61, *p* < 0.001). *p < 0.05, **p < 0.01, ***p < 0.001, ns, no significance

The results of ssGSEA suggested that almost immune cell subpopulations and functions were significantly different among the low‐ and high‐risk groups (Figure 8A,B). Given the importance of ICI in melanoma, we explored the difference in the expression of immune target genes between the two groups. We found the expression of PDCD‐1 (PD‐1), CD247 (PD‐L1), CTLA4, and LAG3 were significantly higher in low‐risk group compared with those in high‐risk group (Figure 8C–F). The results of the spearman correlation analysis showed that the risk score was negatively correlated with PDCD‐1 (*R* = −0.59, *p* < 0.001, Figure 8G), CD247 (*R* = −0.67, *p* < 0.001, Figure 8H), CTLA4 (*R* = −0.49, *p* <0.001, Figure 8I), and LAG3 (*R* = −0.61, *p* < 0.001, Figure 8J).

### 3.7 The qPCR analyses

**FIGURE 9:**
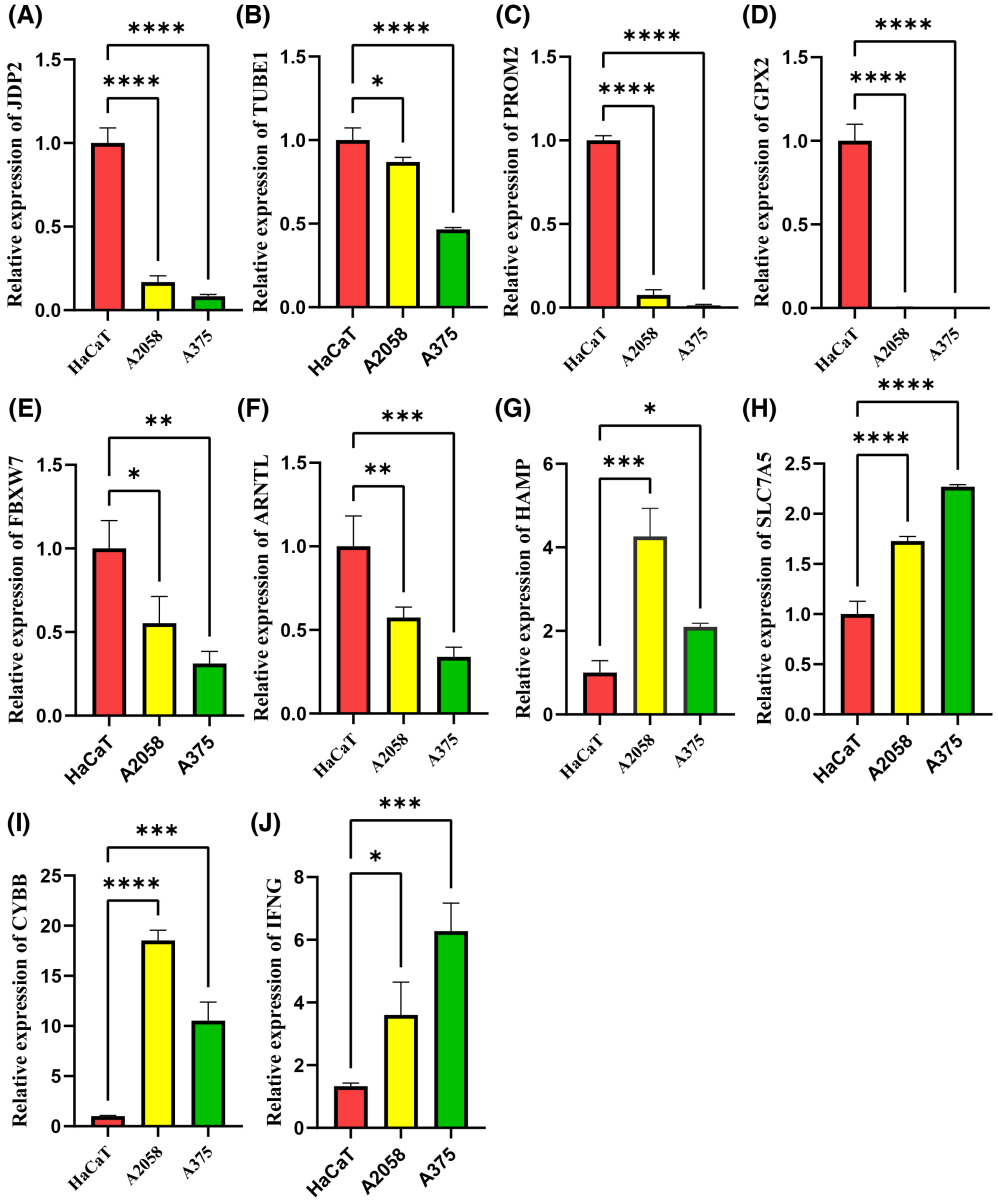
The differential expression of ferroptosis‐related genes was detected by qPCR. (A–F) Compared with the HaCaT cell line, the mRNA of JDP2, TUBE1, PROM2, GPX2, FBXW7, and ARNTL were significantly lower in the A2058 and A375 cell lines, (G–J) while the mRNA of HAMP, SLC7A5, CYBB, and IFNG were significantly higher in the A2058 and A375 cell lines. *p < 0.05, **p < 0.01, ***p < 0.001, ****p < 0.0001

In order to further verify the results of 10 differential expressed genes in the signature, qPCR was used to detect these genes expression at the mRNA level in vitro. These results of qPCR confirmed that the expression of JDP2, TUBE1, PROM2, GPX2, FBXW7, and ARNTL genes in A2058 and A375 cell lines (SKCM tumor cells) were significantly lower than that in the HaCaT cell line (normal skin cells) (Figure 9A–F). Conversely, the expression of HAMP, SLC7A5, IFNG, and CYBB genes in A2058 and A375 cell lines were significantly higher than that in the HaCaT cell line (Figure 9G–J). These findings were consistent with the differential expression of FRGs genes between normal and tumors tissues, further validating the accuracy and reliability of this signature model.

## 4 DISCUSSION

This study identified FRGs by comparing RNA‐seq data from normal samples of GTEx and tumor samples of TCGA. Thirteen differentially expressed FRGs were identified in TCGA‐SKCM cohort by univariate Cox regression analysis, and 10 OS‐related FRGs in SKCM were selected by LASSO. There were significant differences in risk scores and survival between the high‐risk and low‐risk groups in the training and validation cohorts, demonstrating the effectiveness and accuracy of the signature in SKCM. GO, KEGG, and GSEA analyses demonstrated that these FRGs were significantly enriched in immune‐related function and immune‐related pathways. Compared with previous prognostic signature of SKCM, this study is the first time to use FRGs for training and validated in an independent cohort. This study aimed to provide more information for future SKCM studies.

Ferroptosis is an iron‐dependent regulatory cell death induced by lipid peroxidation.^29^ Ferroptosis differed from autophagy, apoptosis, and necrosis regarding cellular, molecular, and biochemical mechanisms and involves the plasma membrane integrity, cytoplasmic and organelle swelling, and moderate chromatin condensation.^30^, ^31^ Ferroptosis has been demonstrated to be involved in the pathophysiological processes of many diseases, such as ischemia–reperfusion injury, neurological disorders, blood diseases, kidney injury, and tumors.^11^

Ferroptosis is used in cancer therapeutics^32^ and regulates signaling pathways in melanoma cells.^33^ In this respect, miR‐9 inhibited RSL3‐ and erastin‐induced ferroptosis, and miR‐9 silencing‐induced ferroptosis in these cells.^34^ miR‐137‐ regulated necroptosis in melanoma cells, and miR‐137 inactivation enhanced the sensitivity of these cells to RSL3‐ and erastin‐induced ferroptosis.^35^ In immunotherapy, activated CD8^+^ T cells enhanced ferroptosis‐mediated lipid peroxidation in tumor cells by downregulating the expression of SLC7A11and SLC3A2 through the release of γ‐IFN.^36^ Melanoma cells depended on oleic acid for freedom from acsl3‐mediated ferroptosis in lymph and increased cell survival and metastatic potential.^19^ These data demonstrate that ferroptosis has great potential of in SKCM treatment and prognosis. Nonetheless, the physiological mechanisms of ferroptosis involved in tumorigenesis and metastasis are unclear.

Some studies identified prognostic FRG signatures by searching public databases. For instance, a signature improved diagnosis and accuracy of prognosis prediction of hepatocellular carcinoma.^37^ A 19‐gene signature accurately predicted the outcome of glioma patients.^20^ However, an FRG‐based prognostic model for SKCM has not been established. The 10‐FRG signature developed in the present study was strongly correlated with the prognosis of SKCM.

The signature contained the genes CYBB, IFNG, FBXW7, ARNTL, PROM2, GPX2, JDP2, SLC7A5, TUBE1, and HAMP and was a strong predictor of OS in training and validation cohort. Differential expression of these genes was validated using qPCR. The genes of HAMP, SLC7A5, CYBB, and IFNG were highly expressed in melanoma cell lines, while JDP2, TUBE1, PROM2, GPX2, FBXW7, and ARNTL were lowly expressed in melanoma cell lines. PROM2, GPX2, PROM2, GPX2, and SLC7A5 were risk factors for OS, while other genes were protective factors. The CYBB and NADPH oxidase genes exhibited sex differential expression in multiple sclerosis.^38^ CYBB was associated with poorer disease‐free survival and OS in melanoma.^39^ IFNG predicted survival and the response to ICIs in melanoma.^40^ FBXW7 is a commonly mutated and inactivated tumor suppressor and was shown to increase resistance to anti‐PD‐1 and improve the clinical response to ICIs in cancer patients.^41^, ^42^, ^43^ The clock gene ARNTL inhibited melanoma cell growth and enhanced immunotherapeutic efficacy by improving effector functions, macrophage mitochondrial metabolism, and redox homeostasis.^44^, ^45^ PROM2 promoted ferroptosis resistance in tumor cells through stimulating the production of ferritin‐containing exosomes and multivesicular bodies and increasing ferritin export.^46^ GPX2 suppressed ferroptosis by silencing lipoxygenases and reducing lipid hydroperoxides.^47^ Although the prognostic value of JDP2, SLC7A5, TUBE1, and HAMP in SKCM is currently unknown, the importance of these four genes should not be underestimated. Our study found that these four genes affected the prognosis of SKCM, which might not be directly administered but was determined by the upstream or downstream of these genes. There is no research on the impact of these four genes on SKCM prognosis, which makes our findings exciting and certainly worthy of further research.

Although ferroptosis has recently been a hot topic of study in tumor, the association between ferroptosis and tumor immunity is incompletely understood. KEGG and GO analyses of DEGs showed that many immune‐related pathways and biological processes were enriched, indicating that ferroptosis associated with tumor immunity. Significant differences in immune response‐activating signal transduction and immune response‐activating cell surface receptor signaling pathway were observed between the low‐ and high‐risk groups. This implied that ferroptotic cells release certain signaling molecules such as lipid mediators to attract T and B cells. GSEA in the HALLMARK collection showed that gene sets for antigen processing and presentation, chemokine signaling pathway, natural killer cell‐mediated cytotoxic city, and Toll‐like receptor signaling pathway were enriched, demonstrating that ferroptosis is involved in cell death, host defense, and innate and adaptive immunity.^48^ Cancers are currently being treated using immunotherapy and iron nanoparticles.^49^, ^50^ Nanoparticles induce ferroptosis by regulating iron and ROS levels and are a novel strategy for cancer therapy.^50^ Immunotherapy has dramatically improved the outcomes of advanced‐stage melanoma patients.^51^ A recent research found that ferroptosis enhanced the antitumor effect of ICIs, even in ICIs‐resistant tumors.^48^ Therefore, a novel FRGs signature were constructed to investigate the potential relationship between ferroptosis and ICIs, and to predict immune‐checkpoint blockade immunotherapy responses. In this study, the FRGs were revealed to be associated with ICIs (PD‐1, PD‐L1, CTLA‐4, and LAG‐3), which indicated that the FRGs signature might be used to predict the response to ICB therapy. In the meantime, the expression levels of these ICIs in low‐risk group were higher compared with high‐risk group. This suggested that FRGs signature could be applied to guide immunotherapy based on predicted expression level of ICIs.

Our study has several limitations. First, this prognostic signature was constructed using public databases. Therefore, multicenter clinical trials are needed to validate the clinical utility of this model. Second, other genes may be good predictors of SKCM prognosis. Third, the association between risk scores and immune responses has not been experimentally demonstrated.

## 5 CONCLUSIONS

Our study identified a signature with 10 ferroptosis‐related genes. The signature was independently correlated with OS in both the training and validation cohorts, improving the accuracy of predicting the prognosis of SKCM. Nonetheless, additional studies are warranted to determine the mechanism underlying the correlation between ferroptosis‐related genes and immunity in SKCM.

## CONFLICT OF INTEREST

The authors declare no conflict of interest in this work.

## AUTHOR CONTRIBUTIONS

Jianghai Chen and Zhuo Wei conceptualized and supervised the study. Shuai Ping, Siyuan Wang, Yingsong Zhao, and JH downloaded and analyzed the data. Shuai Ping, Guanglei Li, and DL wrote the original draft. JH and ZW revised final draft.

## ETHICS STATEMENT

All data of our study were not involved with animal and human experiments and required no ethical approval.

## Data Availability

The original data of the study could be required from the corresponding author on reasonable request.

## References

[1] Siegel RL , Miller KD , Fuchs HE , Jemal A . Cancer Statistics, 2021. CA Cancer J Clin. 2021;71(1):7‐33. doi:10.3322/caac.21654 33433946

[2] Bomar L , Senithilnathan A , Ahn C . Systemic therapies for advanced melanoma. Dermatol Clin. 2019;37(4):409‐423. doi:10.1016/j.det.2019.05.001 31466582

[3] Leonardi GC , Candido S , Falzone L , Spandidos DA , Libra M . Cutaneous melanoma and the immunotherapy revolution (review). Int J Oncol. 2020;57(3):609‐618. doi:10.3892/ijo.2020.5088 32582963 PMC7384846

[4] Villani A , Scalvenzi M , Fabbrocini G , Ocampo‐Candiani J , Ocampo‐Garza SS . Looking into a better future: novel therapies for metastatic melanoma. Dermatol Ther (Heidelb). 2021;11(3):751‐767. doi:10.1007/s13555-021-00525-9 33866515 PMC8163929

[5] Guerrisi A , Loi E , Ungania S , et al. Novel cancer therapies for advanced cutaneous melanoma: the added value of radiomics in the decision making process‐a systematic review. Cancer Med. 2020;9(5):1603‐1612. doi:10.1002/cam4.2709 31951322 PMC7050080

[6] Falzone L , Salomone S , Libra M . Evolution of cancer pharmacological treatments at the turn of the third millennium. Front Pharmacol. 2018;9:1300. doi:10.3389/fphar.2018.01300 30483135 PMC6243123

[7] Cronin KA , Lake AJ , Scott S , et al. Annual report to the nation on the status of cancer, part I: national cancer statistics. Cancer. 2018;124(13):2785‐2800. doi:10.1002/cncr.31551 29786848 PMC6033186

[8] Weinstein D , Leininger J , Hamby C , Safai B . Diagnostic and prognostic biomarkers in melanoma. J Clin Aesthet Dermatol. 2014;7(6):13‐24. https://pubmed.ncbi.nlm.nih.gov/25013535 PMC4086529 25013535

[9] Miller R , Walker S , Shui I , Brandtmüller A , Cadwell K , Scherrer E . Epidemiology and survival outcomes in stages II and III cutaneous melanoma: a systematic review. Melanoma Manag. 2020;7(1):MMT39. doi:10.2217/mmt-2019-0022 32399177 PMC7212505

[10] Spagnolo F , Picasso V , Lambertini M , Ottaviano V , Dozin B , Queirolo P . Survival of patients with metastatic melanoma and brain metastases in the era of MAP‐kinase inhibitors and immunologic checkpoint blockade antibodies: a systematic review. Cancer Treat Rev. 2016;45:38‐45. doi:10.1016/j.ctrv.2016.03.003 26975020

[11] Li J , Cao F , Yin H‐L , et al. Ferroptosis: past, present and future. Cell Death Dis. 2020;11(2):88. doi:10.1038/s41419-020-2298-2 32015325 PMC6997353

[12] Wu Y , Yu C , Luo M , et al. Ferroptosis in cancer treatment: another way to Rome. Front Oncol. 2020;10:571127. doi:10.3389/fonc.2020.571127 33102227 PMC7546896

[13] Bebber CM , Müller F , Prieto Clemente L , Weber J , von Karstedt S . Ferroptosis in cancer cell biology. Cancers (Basel). 2020;12(1). doi:10.3390/cancers12010164 PMC7016816 31936571

[14] Liang C , Zhang X , Yang M , Dong X . Recent Progress in ferroptosis inducers for cancer therapy. Adv Mater Weinheim. 2019;31(51):e1904197. doi:10.1002/adma.201904197 31595562

[15] Hassannia B , Vandenabeele P , Vanden BT . Targeting ferroptosis to iron out cancer. Cancer Cell. 2019;35(6):830‐849. doi:10.1016/j.ccell.2019.04.002 31105042

[16] Jiang M , Qiao M , Zhao C , Deng J , Li X , Zhou C . Targeting ferroptosis for cancer therapy: exploring novel strategies from its mechanisms and role in cancers. Transl Lung Cancer Res. 2020;9(4):1569‐1584. doi:10.21037/tlcr-20-341 32953528 PMC7481593

[17] Ooko E , Saeed MEM , Kadioglu O , et al. Artemisinin derivatives induce iron‐dependent cell death (ferroptosis) in tumor cells. Phytomedicine. 2015;22(11):1045‐1054. doi:10.1016/j.phymed.2015.08.002 26407947

[18] Yamaguchi H , Hsu JL , Chen C‐T , et al. Caspase‐independent cell death is involved in the negative effect of EGF receptor inhibitors on cisplatin in non‐small cell lung cancer cells. Clin Cancer Res. 2013;19(4):845‐854. doi:10.1158/1078-0432.CCR-12-2621 23344263 PMC3703145

[19] Ubellacker JM , Tasdogan A , Ramesh V , et al. Lymph protects metastasizing melanoma cells from ferroptosis. Nature. 2020;585(7823):113‐118. doi:10.1038/s41586-020-2623-z 32814895 PMC7484468

[20] Liu H‐J , Hu H‐M , Li G‐Z , et al. Ferroptosis‐related gene signature predicts glioma cell death and glioma patient progression. Front Cell Dev Biol. 2020;8:538. doi:10.3389/fcell.2020.00538 32733879 PMC7363771

[21] Liang J‐Y , Wang D‐S , Lin H‐C , et al. A novel ferroptosis‐related gene signature for overall survival prediction in patients with hepatocellular carcinoma. Int J Biol Sci. 2020;16(13):2430‐2441. doi:10.7150/ijbs.45050 32760210 PMC7378635

[22] Carithers LJ , Ardlie K , Barcus M , et al. A novel approach to high‐quality postmortem tissue procurement: the GTEx project. Biopreserv Biobank. 2015;13(5):311‐319. doi:10.1089/bio.2015.0032 26484571 PMC4675181

[23] Zhou N , Bao J . FerrDb: a manually curated resource for regulators and markers of ferroptosis and ferroptosis‐disease associations. Database (Oxford). 2020;2020:311‐319. doi:10.1093/database/baaa021 PMC7100629 32219413

[24] Gui J , Li H . Penalized cox regression analysis in the high‐dimensional and low‐sample size settings, with applications to microarray gene expression data. Bioinformatics (Oxford, England). 2005;21(13):3001‐3008. https://pubmed.ncbi.nlm.nih.gov/15814556 10.1093/bioinformatics/bti422 15814556

[25] Wu M , Li X , Zhang T , Liu Z , Zhao Y . Identification of a nine‐gene signature and establishment of a prognostic nomogram predicting overall survival of pancreatic cancer. Front Oncol. 2019;9:996. doi:10.3389/fonc.2019.00996 31612115 PMC6776930

[26] Goodman A , Patel SP , Kurzrock R . PD‐1‐PD‐L1 immune‐checkpoint blockade in B‐cell lymphomas. Nat Rev Clin Oncol. 2017;14(4):203‐220. doi:10.1038/nrclinonc.2016.168 27805626

[27] Nishino M , Ramaiya NH , Hatabu H , Hodi FS . Monitoring immune‐checkpoint blockade: response evaluation and biomarker development. Nat Rev Clin Oncol. 2017;14(11):655‐668. doi:10.1038/nrclinonc.2017.88 28653677 PMC5650537

[28] Tirosh I , Izar B , Prakadan SM , et al. Dissecting the multicellular ecosystem of metastatic melanoma by single‐cell RNA‐seq. Science (New York, NY). 2016;352(6282):189‐196. doi:10.1126/science.aad0501 PMC4944528 27124452

[29] Stockwell BR , Friedmann Angeli JP , Bayir H , et al. Ferroptosis: a regulated cell death nexus linking metabolism, redox biology, and disease. Cell. 2017;171(2):273‐285. doi:10.1016/j.cell.2017.09.021 28985560 PMC5685180

[30] Dixon SJ , Lemberg KM , Lamprecht MR , et al. Ferroptosis: an iron‐dependent form of nonapoptotic cell death. Cell. 2012;149(5):1060‐1072. doi:10.1016/j.cell.2012.03.042 22632970 PMC3367386

[31] Tao N , Li K , Liu J . Molecular mechanisms of ferroptosis and its role in pulmonary disease. Oxid Med Cell Longev. 2020;2020:9547127‐9547112. doi:10.1155/2020/9547127 32685102 PMC7338975

[32] Mou Y , Wang J , Wu J , et al. Ferroptosis, a new form of cell death: opportunities and challenges in cancer. J Hematol Oncol. 2019;12(1):34. doi:10.1186/s13045-019-0720-y 30925886 PMC6441206

[33] Ashrafizadeh M , Mohammadinejad R , Tavakol S , Ahmadi Z , Roomiani S , Katebi M . Autophagy, anoikis, ferroptosis, necroptosis, and endoplasmic reticulum stress: potential applications in melanoma therapy. J Cell Physiol. 2019;234(11):19471‐19479. doi:10.1002/jcp.28740 31032940

[34] Zhang K , Wu L , Zhang P , et al. miR‐9 regulates ferroptosis by targeting glutamic‐oxaloacetic transaminase GOT1 in melanoma. Mol Carcinog. 2018;57(11):1566‐1576. doi:10.1002/mc.22878 30035324

[35] Luo M , Wu L , Zhang K , et al. miR‐137 regulates ferroptosis by targeting glutamine transporter SLC1A5 in melanoma. Cell Death Differ. 2018;25(8):1457‐1472. doi:10.1038/s41418-017-0053-8 29348676 PMC6113319

[36] Wang W , Green M , Choi JE , et al. CD8 T cells regulate tumour ferroptosis during cancer immunotherapy. Nature. 2019;569(7755):270‐274. doi:10.1038/s41586-019-1170-y 31043744 PMC6533917

[37] Tang B , Zhu J , Li J , et al. The ferroptosis and iron‐metabolism signature robustly predicts clinical diagnosis, prognosis and immune microenvironment for hepatocellular carcinoma. Cell Commun Signal. 2020;18(1):174. doi:10.1186/s12964-020-00663-1 33115468 PMC7592541

[38] Cardamone G , Paraboschi EM , Soldà G , Duga S , Saarela J , Asselta R . Genetic association and altered gene expression of in multiple sclerosis patients. Biomedicine. 2018;6(4). doi:10.3390/biomedicines6040117 PMC6315774 30567305

[39] Zhu J , Hao S , Zhang X , Qiu J , Xuan Q , Ye L . Integrated bioinformatics analysis exhibits pivotal exercise‐induced genes and corresponding pathways in malignant melanoma. Front Genet. 2020;11:637320. doi:10.3389/fgene.2020.637320 33679872 PMC7930906

[40] Karachaliou N , Gonzalez‐Cao M , Crespo G , et al. Interferon gamma, an important marker of response to immune checkpoint blockade in non‐small cell lung cancer and melanoma patients. Ther Adv Med Oncol. 2018;10:1758834017749748. doi:10.1177/1758834017749748 29383037 PMC5784541

[41] Zhao E , Maj T , Kryczek I , et al. Cancer mediates effector T cell dysfunction by targeting microRNAs and EZH2 via glycolysis restriction. Nat Immunol. 2016;17(1):95‐103. doi:10.1038/ni.3313 26523864 PMC4684796

[42] Gstalder C , Liu D , Miao D , et al. Inactivation of impairs dsRNA sensing and confers resistance to PD‐1 blockade. Cancer Discov. 2020;10(9):1296‐1311. doi:10.1158/2159-8290.CD-19-1416 32371478 PMC8802534

[43] Aydin IT , Melamed RD , Adams SJ , et al. FBXW7 mutations in melanoma and a new therapeutic paradigm. J Natl Cancer Inst. 2014;106(6):dju107. doi:10.1093/jnci/dju107 24838835 PMC4081626

[44] Kiessling S , Beaulieu‐Laroche L , Blum ID , et al. Enhancing circadian clock function in cancer cells inhibits tumor growth. BMC Biol. 2017;15(1):13. doi:10.1186/s12915-017-0349-7 28196531 PMC5310078

[45] Alexander RK , Liou Y‐H , Knudsen NH , et al. Bmal1 integrates mitochondrial metabolism and macrophage activation. Elife. 2020;9. doi:10.7554/eLife.54090 PMC7259948 32396064

[46] Brown CW , Amante JJ , Chhoy P , et al. Prominin2 drives ferroptosis resistance by stimulating iron export. Dev Cell. 2019;51(5):575‐586.e4. doi:10.1016/j.devcel.2019.10.007 31735663 PMC8316835

[47] Brigelius‐Flohé R , Flohé L . Regulatory phenomena in the glutathione peroxidase superfamily. Antioxid Redox Signal. 2020;33(7):498‐516. doi:10.1089/ars.2019.7905 31822117

[48] Tang R , Xu J , Zhang B , et al. Ferroptosis, necroptosis, and pyroptosis in anticancer immunity. J Hematol Oncol. 2020;13(1):110. doi:10.1186/s13045-020-00946-7 32778143 PMC7418434

[49] Büttner N , Schmidt N , Thimme R . Perspectives of immunotherapy in hepatocellular carcinoma (HCC). Zeitschrift Fur Gastroenterologie. 2016;54(12):1334‐1342. https://pubmed.ncbi.nlm.nih.gov/27936483 27936483 10.1055/s-0042-120417

[50] Tarangelo A , Dixon SJ . Nanomedicine: an iron age for cancer therapy. Nat Nanotechnol. 2016;11(11):921–922. doi:10.1038/nnano.2016.199 27668797 PMC6091215

[51] Luke JJ , Flaherty KT , Ribas A , Long GV . Targeted agents and immunotherapies: optimizing outcomes in melanoma. Nat Rev Clin Oncol. 2017;14(8):463‐482. doi:10.1038/nrclinonc.2017.43 28374786

